# Underdominant KCC3b R31I association with blood sodium concentration in domestic sheep suggests role in oligomer function

**DOI:** 10.1111/age.12585

**Published:** 2017-07-27

**Authors:** Stephen N. White, Ryan D. Oliveira, Michelle R. Mousel, Michael V. Gonzalez, Margaret A. Highland, Maria K. Herndon, J. Bret Taylor, Donald P. Knowles

**Affiliations:** ^1^ Animal Disease Research USDA‐ARS Pullman WA 99164 USA; ^2^ Department Veterinary Microbiology and Pathology Washington State University Pullman WA 99164 USA; ^3^ Center for Reproductive Biology Washington State University Pullman WA 99164 USA; ^4^ School for Global Animal Health Washington State University Pullman WA 99164 USA; ^5^ Washington Animal Disease Diagnostic Laboratory Washington State University Pullman WA 99164 USA; ^6^ Range Sheep Production Efficiency Research USDA‐ARS, Dubois ID 83423 USA; ^7^ Present address: Perelman School of Medicine University of Pennsylvania Philadelphia PA 19104 USA

KCC3 and KCC1 are potassium chloride transporters encoded by *SLC12A6* and *SLC12A4* respectively with partially overlapping function,^1^ and KCC3 knockout mice exhibit hypertension.^2^ Two KCC3 isoforms differ by alternate promoters and first coding exons^3^: KCC3a is widely expressed and KCC3b is highly expressed in kidney proximal convoluted tubule.^4^ We genotyped KCC3 and KCC1 amino acid substitutions using Taqman assays (Table S1) in 307 Suffolk, Rambouillet, Polypay and Columbia sheep. Whole blood sodium and potassium concentrations were determined by atomic absorbance spectrometry. Association was determined by mixed models in sas 9.2 (SAS Institute, Cary, NC, USA) with breed and genotype for variant of interest as fixed effects and sire nested within breed treated as random. Preliminary testing showed age in years was not related to ion concentrations (*P *>* *0.05), so age was not included in the models. No KCC3a or KCC1 substitutions (Table S2) were associated with blood potassium or sodium (all *P *>* *0.05). KCC3b R31I is a charged substitution located in a conserved motif (Table S3), and although it was not associated with potassium (*P *>* *0.05), it was associated with blood sodium in an underdominant manner (Table 1) whereby sodium was significantly higher in both homozygotes than in heterozygotes (*P *<* *0.05). The sodium association is interesting because: (i) KCC3 is known to transport only potassium and chloride, not sodium^5^ and (ii) KCC3a interacts with the sodium–potassium pump, whereas KCC3b does not.^6^ Possible mechanisms include: (i) R31I alteration of KCC3b sodium affinity, (ii) KCC3b oligomer formation with sodium transporters^7^ or (iii) indirect influence, e.g. by impairing sodium–potassium pump efficiency through potassium availability.^8^ Regardless of the specific mechanism, the underdominant pattern suggests allelic incompatibility^9^ such as dimer impairment. Because KCC3 functions as a homodimer,^10^ R31I may interfere with KCC3b dimer function in regulating sodium concentration. To our knowledge, this is the first report of a KCC3 variant associated with blood sodium. These data suggest further study of coordinated function between KCC3 and sodium transport, and that sheep with KCC3b R31I may serve as a biomedical model.

**Table 1:** Adjusted mean blood sodium concentrations by *SLC12A6* (KCC3b) R31I genotype

Genotype	*n*	Adjusted mean sodium conc. (mm)
II^a^	47	128.9
IR^b^	109	120.6
RR^a^	140	126.1

Tukey‐Kramer pairwise comparisons: II^a^ > IR^b^ (*P *=* *0.008); RR^a^ > IR^b^ (*P *=* *0.02); RR^a^ not different than II^a^ (*P *=* *0.37).

## Conflict of interest

The authors declare no conflicts of interest exist.

## Supporting information


**Table S1** Genotyping reagents.

**Table S2** Breed allele frequencies.

**Table S3** Amino acid alignment of mammal KCC3b highlighting R31I.
