# Supplementary material for: Underdominant KCC3b R31I association with blood sodium concentration in domestic sheep suggests role in oligomer function
Source: Anim Genet. 2017 Jul 27;48(5):626–7. doi: 10.1111/age.12585 (PMC5638067; doi:10.1111/age.12585)
Supplement: Supplementary file 1 — Table S1 Genotyping reagents. [file AGE-48-626-s001.pdf]

Table S1. Genotyping reagents.

dbSNP	Variant Name	Annotation	Amplification Primer 1[6]	Amplification Primer 2	Probe 1[7]	Probe 2[8]
rs426648951	g.25292281T>G[1]	KCC3b[3] R31I*	GCAGCTTCCGGCTCTCA	GTCCGTCTGAATGCTGA	AGCAGAAAGAAAA	CAGCAGAAATAAAA
rs599656845	g.25271483C>G[1]	KCC3a[4] R42G	CCGGACCTCAGCTCTCG	ACACTTTCGCGGGAGCT	ATCCAGTTCCCGAG	CCAGTTCCGGAGTA
rs401017839	g.25346233G>A[1]	KCC3a[4] V555M	ACGTCCCCACTCTGATTTCT	GGCCAAGATAAGGTTCC	TTGGAGATGCTATG	TGGAGATGCTGTGA
rs595415030	g.25357125A>T[1]	KCC3a[4] Q1042L	TGACGAGGATGACGAGACA	TGTCTTTCGTCCACGTC	CACCTTCTCCTGAT	CCTTCTCCAGATAA
rs411467556	g.34680944A>G[2]	KCC1[5] M290T	TGGCCACTGTCTCGTTGTC	ACTGTCCCGGGACCAGT	ACCACAGTCATCTT	ACCACAGTCGTCTT
rs407003706	g.34680165A>G[2]	KCC1[5] V377A	CAGCACGGTGAAGGATGTG	GACGCCCTTGGCTTGAA	CAATGTCAGCCACC	ATGTCAGCCGCCAC
rs603768252	g.34678614C>T[2]	KCC1[5] G510S	CGTCGGTTCGCCATTCG	TCGCTTCTGACCCCATT	ACAGGTTTTCGGCC	ACAGGTTTTCAGCC
ss213751737	g.34675697G>A[2]	KCC1[5] T767I	CCTTCACCTTCTCAATCTCC	CCTTCTGACCCGGGTAT	CCCTACAGATAATC	CCTACAGACAATCA

[1]Variant names in *SLC12A6* which encodes KCC3 are in reference to NW_014639016.1 in OARv4.0.
[2]Variant names in *SLC12A4* which encodes KCC1 are in reference to NW_014639023.1 in OARv4.0.
[3]KCC3b amino acid positions are in reference to KCC3b transcript (GenBank XM_004010416).
[4]KCC3a amino acid positions are in reference to KCC3a transcript (GenBank XM_012181134).
[5]KCC1 amino acid positions are in reference to KCC1 transcript (GenBank XM_015099947).
[6]Order numbers (Applied Biosystems, Inc.) for reagents described in sequential order: AHUAPR9, ANYMJJZ, AHABF7J, ANZTD4X, AN2W7T2, AHWSJBZ, AN322AT, AN2W7PV, AHVJNYG
[7]Probe labeled with VIC dye.
[8]Probe labeled with FAM dye.
*KCC3b R31I identified showing underdominant association with sheep blood sodium concentration.
