# Supplementary material for: Underdominant KCC3b R31I association with blood sodium concentration in domestic sheep suggests role in oligomer function
Source: Anim Genet. 2017 Jul 27;48(5):626–7. doi: 10.1111/age.12585 (PMC5638067; doi:10.1111/age.12585)
Supplement: Supplementary file 2 — Table S2 Breed allele frequencies. [file AGE-48-626-s002.pdf]

Table S2. Breed allele frequencies.

					Breed Allele Frequency (%)			
**dbSNP**	**Gene**	**Variant Name**	**Functional Consequence**	**Minor Allele [amino acid (nucleotide)]**	**Suffolk**	**Rambouillet**	**Polypay**	**Columbia**
rs426648951	*SLC12A6*	g.25292281T>G[1]	KCC3b[3] R31I*	I (T)	33.5%	51.5%	17.6%	29.6%
rs599656845	*SLC12A6*	g.25271483C>G[1]	KCC3a[4] R42G	G (G)	0.0%	0.0%	0.0%	0.0%
rs401017839	*SLC12A6*	g.25346233G>A[1]	KCC3a[4] V555M	M (A)	0.0%	7.1%	4.9%	5.6%
rs595415030	*SLC12A6*	g.25357125A>T[1]	KCC3a[4] Q1042L	L (T)	0.0%	0.0%	0.0%	0.0%
rs411467556	*SLC12A4*	g.34680944A>G[2]	KCC1[5] M290T	T (G)	34.2%	41.9%	28.6%	31.5%
rs407003706	*SLC12A4*	g.34680165A>G[2]	KCC1[5] V377A	A (G)	0.0%	0.0%	0.5%	0.0%
rs603768252	*SLC12A4*	g.34678614C>T[2]	KCC1[5] G510S	S (T)	0.0%	0.0%	0.0%	0.0%
ss2137517374	*SLC12A4*	g.34675697G>A[2]	KCC1[5] T767I	I (T)	0.6%	2.8%	4.7%	0.0%

[1]Variant names in *SLC12A6* which encodes KCC3 are in reference to NW_014639016.1 in OARv4.0.
[2]Variant names in *SLC12A4* which encodes KCC1 are in reference to NW_014639023.1 in OARv4.0.
[3]KCC3b amino acid positions are in reference to KCC3b transcript (GenBank XM_004010416).
[4]KCC3a amino acid positions are in reference to KCC3a transcript (GenBank XM_012181134).
[5]KCC1 amino acid positions are in reference to KCC1 transcript (GenBank XM_015099947).
*KCC3b R31I identified showing underdominant association with sheep blood sodium concentration.
