# Supplementary material for: Underdominant KCC3b R31I association with blood sodium concentration in domestic sheep suggests role in oligomer function
Source: Anim Genet. 2017 Jul 27;48(5):626–7. doi: 10.1111/age.12585 (PMC5638067; doi:10.1111/age.12585)
Supplement: Supplementary file 3 — Table S3 Amino acid alignment of mammal KCC3b highlighting R31I. [file AGE-48-626-s003.pdf]

Table S3. Amino acid alignment of mammal KCC3b highlighting R31I.

**Species**	**Accession**	**Alignment**																			
**Sheep**	R31 substituted XP_00401465	E	G	E	P	S	A	A	E	R	K	A	P	I	Q	H	S	D	G	P	D
**Sheep**	XP_004010465	E	G	E	P	S	A	A	E	I	K	A	P	I	Q	H	S	D	G	P	D
**Goat**	XP_017909992	E	G	E	P	S	A	A	E	I	K	A	P	I	Q	H	S	D	G	P	D
**Cow**	XP_005211554	E	G	E	P	S	L	A	E	I	K	A	P	I	Q	H	S	D	E	P	D
**Water buffalo**	XP_006056078	E	G	E	P	S	L	A	E	I	K	A	P	I	Q	H	S	D	E	P	D
**Camel**	XP_006194902	E	R	E	P	S	L	A	E	I	K	A	P	I	Q	H	S	D	E	P	D
**Weddell seal**	XP_006730697	E	G	Q	P	S	L	A	E	I	K	A	L	I	Q	H	S	D	E	P	D
**Dog**	XP_856607	E	G	E	P	S	L	A	E	R	R	A	L	I	Q	H	L	D	E	P	D
**Cat**	XP_011281389	E	G	A	T	S	L	A	D	I	K	A	L	I	Q	H	S	D	E	L	D
**Human**	NP_005126	E	-	-	P	S	L	A	D	I	K	A	R	I	Q	D	S	D	E	P	D
**Mouse**	NP_598409	E	-	-	P	S	S	A	E	V	K	A	R	I	Q	D	P	Q	E	P	D
**Charged versus**	**Polar versus Nonpolar**	**C**	**CN**	**CPN**	**PN**	**P**	**PN**	**N**	**C**	**CN***	**C**	**N**	**CN**	**N**	**P**	**C**	**PN**	**CP**	**CN**	**N**	**C**

*Homologous residues to sheep KCC3b 29-33 show a particularly conserved pattern for 2 amino acid residues on either side of R31I, which is Alanine-negatively Charged-(Charged/Nonpolar-31)-positively Charged-Alanine.
